# Rutaecarpine Increases Anticancer Drug Sensitivity in Drug-Resistant Cells through MARCH8-Dependent ABCB1 Degradation

**DOI:** 10.3390/biomedicines9091143

**Published:** 2021-09-02

**Authors:** Tingting Zou, Cheng Zeng, Junyan Qu, Xiaohua Yan, Zhenghong Lin

**Affiliations:** 1 School of Life Sciences, Chongqing University, Chongqing 401331, China; tingtzou1997@163.com (T.Z.); cqzengcheng@163.com (C.Z.); qujunyan1229@yeah.net (J.Q.); 2 Department of Biochemistry and Molecular Biology, School of Basic Medical Sciences, Nanchang University, Nanchang 330006, China

**Keywords:** rutaecarpine, multidrug resistance (MDR), ABCB1, MARCH8, ubiquitination

## Abstract

The overexpression of adenosine triphosphate (ATP)-binding cassette (ABC) subfamily B member 1 (ABCB1; P-glycoprotein; MDR1) in some types of cancer cells is one of the mechanisms responsible for the development of multidrug resistance (MDR), which leads to the failure of chemotherapy. Therefore, it is important to inhibit the activity or reduce the expression level of ABCB1 to maintain an effective intracellular level of chemotherapeutic drugs. In this study, we found that rutaecarpine, a bioactive alkaloid isolated from *Evodia Rutaecarpa*, has the capacity to reverse ABCB1-mediated MDR. Our data indicated that the reversal effect of rutaecarpine was related to the attenuation of the protein level of ABCB1. Mechanistically, we demonstrated that ABCB1 is a newly discovered substrate of E3 ubiquitin ligase membrane-associated RING-CH 8 (MARCH8). MARCH8 can interact with ABCB1 and promote its ubiquitination and degradation. In short, rutaecarpine increased the degradation of ABCB1 protein by upregulating the protein level of MARCH8, thereby antagonizing ABCB1-mediated MDR. Notably, the treatment of rutaecarpine combined with other anticancer drugs exhibits a therapeutic effect on transplanted tumors. Therefore, our study provides a potential chemotherapeutic strategy of co-administrating rutaecarpine with other conventional chemotherapeutic agents to overcome MDR and improve therapeutic effect.

## 1. Introduction

In countries of all income levels, cancer continues to be a leading cause of global death and the most important barrier to increasing life expectancy. Lung cancer and breast cancer are the most frequent cancers and the leading causes of cancer death among males and females, respectively [1]. Despite major breakthroughs in cancer treatment, chemotherapy is still the main treatment for most cancers in the clinic [2]. In recent years, many studies have been devoted to developing new anticancer drugs and optimizing treatment, but the therapeutic efficacy has not been significantly improved. In most instances, anticancer drugs become ineffective in cancer treatment, and this is thought to be caused by acquiring drug resistance in tumor cells [3] or existing cancer stem cells in the tumor microenvironment [4]. Therefore, multidrug resistance (MDR) is recognized as the main obstacle to the successful treatment of cancers [5,6]. MDR occurs by a variety of molecular mechanisms, including increased intracellular drug efflux [7], drug inactivation, altered DNA damage repair [8], oncogene mutations, and the upregulation of drug metabolism-related enzymes. ABC transporter-mediated drug resistance is considered to be one of the important mechanisms for tumor cells to acquire MDR [9,10].

The efflux pump proteins mediating MDR in human cancers belong to the ATP-binding cassette (ABC) superfamily of proteins. ABC transporters belong to one of the largest families of membrane protein complexes, which consists of seven subfamilies from A to G, mediating diverse ATP-driven transport process [11,12]. They contain a pair of conserved cytoplasmic domains termed ATP-binding cassettes or nucleotide-binding domains (NBDs) and two transmembrane domains (TMDs) that are embedded in the membrane bilayer [13]. ABCB1, ABCG2 (breast cancer resistance protein, BCRP), and ABCC1 (multidrug resistance-associated protein 1, MRP1) are the major contributors to the efflux of the chemotherapeutic drugs among ABC transporters [7,14,15]. It is difficult to demonstrate the importance of drug efflux pumps in MDR cancers. One reason for this is that multiple resistance mechanisms are commonly upregulated in tandem in many MDR cells [10]. ABCB1 expression has been frequently observed in some human tumors at relapse after chemotherapy, including acute lymphocytic leukemia (ALL), acute nonlymphocytic leukemia (ANLL), breast cancer, and neuroblastoma. ABCB1 RNA levels were also usually elevated in some types of clinically refractory tumors untreated with chemotherapeutic drugs, such as those derived from the colon, kidney, adrenal gland, liver, and pancreas, as well as in carcinoid tumors, chronic myelogenous leukemia in blast crisis, and cell lines of non-small cell lung cancer (NSCLC) with neuroendocrine properties [16,17]. The well-known transport substrates of ABCB1 are paclitaxel, adriamycin, colchicine, vincristine, and others [18]. In fact, in the treatment of ovarian cancer with paclitaxel, ABCB1 overexpression was reported to correlate inversely with the probability of survival [19]. Therefore, the application of combining ABCB1 inhibitors and anticancer drugs is considered to be a promising strategy to avoid ABCB1-mediated drug efflux [18,20]. Recently, some tyrosine kinase inhibitors (TKIs) such as erdafitinib, midostaurin, tepotinib, and glesatinib have been shown to antagonize ABCB1-mediated MDR by inhibiting its activity [21,22,23,24]. However, no ABCB1 inhibitors have been approved by the Food and Drug Administration (FDA) for clinical treatment due to safety concerns or the lack of clinical data.

In addition, previous studies have shown that ABCB1 is regulated by E3 ubiquitin ligase NEDD4-1, FBXO15, and FBXO211 [25,26,27]. Therefore, targeting the ubiquitin pathway is a new strategy against ABCB1. Membrane-associated RING-CH 8 (MARCH8) is one of 11 members of the MARCH family of RING-finger E3 ubiquitin ligases, which comprises an N-terminal cytoplasmic tail (CT) domain, two transmembrane (TM) domains, between which a short ectodomain is located, and a C-terminal CT domain [28,29]. MARCH8 is located on endosomes and the plasma membrane and downregulates a variety of cellular transmembrane proteins, such as MHC-II [30], CD86 [31], CD81 [32], TRAIL receptor 1 [33], IL-1 receptor accessory protein [34], and HIV-1 envelope glycoproteins from the cell surface [35].

Rutaecarpine (Rut) is a bioactive alkaloid isolated from *Evodia rutaecarpa* (Wu Zhu Yu), a traditional Chinese herb that is clinically used in China to treat headaches, abdominal pain, postpartum hemorrhage, and dysentery [36]. As one of the most representative alkaloids of *Evodia rutaecarpa*, rutaecarpine has shown a variety of pharmacological actions such as anti-thrombotic, anticancer, anti-inflammatory, and analgesic activities [37]. In recent years, many studies have demonstrated that rutaecarpine has great potential for the treatment of colitis, liver disease, acute pancreatitis, diabetes, and Alzheimer’s disease. Interestingly, our study found that rutaecarpine also has great potential in antagonizing drug resistance.

In this study, we proved for the first time that: (1) Rutaecarpine can downregulate the protein level of ABCB1, and its combined use with anticancer drugs can induce the apoptosis of drug-resistant cells; (2) Mechanistically, ABCB1 is a newly discovered substrate for E3 ubiquitin ligase MARCH8. MARCH8 could interact with ABCB1 and promote its ubiquitination and degradation. The expression level of MARCH8 was increased after treatment with rutaecarpine, thereby promoting ABCB1 degradation. In conclusion, we demonstrated that rutaecarpine could increase anticancer drug sensitivity in drug-resistant cells through MARCH8-dependent ABCB1 degradation.

## 2. Materials and Methods

### 2.1. Chemicals and Reagents

Rutaecarpine, paclitaxel, adriamycin, colchicine, and cisplatin were purchased from Chengdu Alfa Biotechnology Co. Ltd. (Chengdu, China). Rhodamine 123, 4′,6-diamidino-2-phenylindole (DAPI), Triton X-100, 4% paraformaldehyde, and enhanced DAB reagent kit were purchased from Solarbio Science & Technology Co. Ltd. (Beijing, China). Tariquidar (ABCB1 inhibitor) was obtained from Beyotime Biotechnology (Shanghai, China). Cell counting kit-8 (CCK8) was purchased from Med Chem Express (MCE). Annexin V-FITC/PI apoptosis detection kit was purchased from Beijing ComWin Biotech Co. Ltd. (CWBIO) (Beijing, China). Fetal bovine serum (FBS) and Dulbecco’s modified Eagle’s Medium (DMEM) were purchased from Corning Incorporated (Corning, New York, NY, USA). The antibodies used for WB, IFC, Co-IP, and IH were raised against the following proteins: ABCB1 (Zen Bioscience, Chengdu, China), β-tubulin (Sigma-Aldrich, St. Louis, MO, USA), Flag (Sigma-Aldrich St. Louis, MO, USA), Myc (Santa Cruz, CA, USA), HA (Santa Cruz, CA, USA), β-actin (Santa Cruz, CA, USA), MARCH8 (BBI Life Science, Shanghai, China), horseradish peroxidase (HRP)-conjugated secondary antibodies to mouse (Beyotime, Shanghai, China), or rabbit (Beyotime, Shanghai, China).

### 2.2. Cell Lines and Cell Culture

Human breast cancer (MCF-7 and MCF-7/ADR) and lung cancer cell lines (A549 and A549/Taxol) were purchased from the Procell Life Science & Technology Co. Ltd. (Wuhan, China). The MCF-7/ADR cell was cultured in medium containing 1 μg/mL adriamycin to maintain its drug-resistant characteristics. The A549/Taxol cell was cultured in medium containing 1 μg/mL paclitaxel to maintain its drug-resistant characteristics. All aforementioned cell lines were maintained in DMEM containing 10% FBS and 1% penicillin/streptomycin at 37 °C in a humidified atmosphere containing 5% CO_2_.

### 2.3. Cytotoxicity and MDR Reversal Experiments

The cytotoxicity of rutaecarpine and MDR reversal experiments were performed by CCK8 assay. Briefly, for rutaecarpine cytotoxicity study, each type of cell was harvested and resuspended before being seeded in a 96-well plate at a final quantity of 5 × 10^3^ cells per well in 100 μL of medium, and was then incubated overnight. Different concentrations of rutaecarpine were added. For reversal study, rutaecarpine and positive control drug were added 2 h prior to incubation with or without anticancer drugs. After 48 h of further incubation, CCK8 was added to each well and the cells were incubated for 2 h at 37 °C. Subsequently, the absorbance value was read at 450 nm. Graphpad Prism software was used to calculate the concentration for 50% inhibition of cell viability (IC_50_) of the anticancer drug. For positive control drug, tariquidar (20 μM) was used as reference inhibitor to reverse ABCB1-mediated MDR. Cisplatin, a non-substrate drug of ABCB1, was used as a negative control anticancer drug for reversal study.

### 2.4. Western Blotting and Immunofluorescence Analysis on ABCB1 Expression

Western blotting analysis was performed as previously described [38]. Briefly, cells were collected and lysed in a radioimmune precipitation assay (RIPA) buffer containing protease inhibitors for about 30 min at 4 °C. The lysates were then centrifuged at 13,000× *g* rpm for 15 min to obtain supernatants without cell debris and nuclei. A total of 10% sodium dodecyl sulfate–polyacrylamide gel electrophoresis (SDS-PAGE) was used to separate the total proteins and thereby transfer proteins to polyvinylidene fluoride (PVDF) membranes, and 5% skim milk powder was applied to block the membranes for 1 h, which were then incubated with the indicated primary antibodies (dilution 1:1000) overnight at 4 °C. After incubating with secondary antibody (dilution 1:5000) for 1 h at room temperature, the bands were visualized using the enhanced chemiluminescence reaction reagent. The resulting protein bands were analyzed using Image J software. 

The immunofluorescence assay was performed as previously described [39]. In brief, after being cultured overnight in 35 mm confocal dish, cells (2 × 10^4^/well) were treated with or without rutaecarpine for 72 h at 20 μM concentration. Then, cells were washed cold phosphate-buffered saline (PBS) twice and fixed in 4% paraformaldehyde for 20 min. Subsequently, after being permeabilized by 0.1% Triton X-100 for 15 min, cells were blocked with BSA (5% with PBS) for 1 h. The presence of ABCB1 was determined using polyclonal antibody ABCB1 (dilution 1:100) for incubation at 4 °C overnight. Cells were washed with iced PBS after each incubation time. Cy3 (Ex = 550 nm, Em = 570 nm) or FITC (Ex = 490 nm, Em = 525 nm) conjugated secondary antibody (dilution 1:1000) was used after washing with iced PBS. DAPI (Ex = 345 nm, Em = 455 nm) was used to counterstain the nuclei. The cells were washed with ice-cold PBS before being imaged. Immunofluorescence images were collected using a Leica laser scanning confocal microscope (LSCM).

### 2.5. RNA Extraction and qRT-PCR

The mRNA expression of ABCB1 was determined by real-time fluorescence quantitative PCR (qRT-PCR) assay as previously described [40]. MCF-7/ADR or A549/Taxol cells (5 × 10^5^/well) were seeded in 60 mm dishes and were treated with various concentrations of rutaecarpine for 48 h. Total RNA was then collected using a TRIzol kit (TaKaRa, Japan) according to the manufacturer’s protocol. cDNA was reverse transcribed from 1 μg of total RNA using a reverse transcription kit (TaKaRa, Japan), and the forward and reverse primers used in the qRT-PCR were as follows: ABCB1 forward, 5′-CAGAGTCAAGGAGCATGGCA-3′; ABCB1 reverse, 5′-TCAGAGTTCACTGGCGCTTT-3′. β-actin was used as a control using the following primers: forward, 5′-CATGTACGTTGCTATCCAGGC-3′; reverse, 5′-CTCCTTAATGTCACGCACGAT-3′. Amplification was performed according to the instructions for the 2×SYBR Green Master Mix (BIO-RAD) using a BIO-RAD C1000^TM^ Thermal Cycler. PCR was performed at 95 °C for 30 s of initial denaturation and then at 95 °C for 30 s of denaturation, 60 °C for 30 s of annealing/extension, 40 cycles. Relative expression levels were calculated using the 2^−ΔΔCt^ method for qRT-PCR.

### 2.6. Rhodamine 123 and Adriamycin Accumulation Assay

Cells were grown in 6-well plates for 12 h and washed twice with phosphate-buffered saline (PBS) before the pre-treatment of rutaecarpine. Cells were treated with or without inhibitors (20 μM, 48 h) before incubation with rhodamine 123 at a dose of 10 μM for an extra 2 h. Tariquidar was used as a positive inhibitor of ABCB1. Fluorescent images were taken under fluorescence microscope. Following that, cells were harvested and cleaned with PBS three times and analyzed with flow cytometry (FCM) as previously described to measure the fluorescence intensity. Similarly, the assay of measuring the accumulation of adriamycin was conducted as rhodamine 123.

### 2.7. Adriamycin Efflux Assay

Cells were incubated into 96-well plates and treated with rutaecarpine or tariquidar (20 μM, 48 h). After being washed twice with PBS, 25 μM adriamycin was added, and incubation was continued for additional 2 h. After various time points (0, 30, 60, 120 min), the cell culture supernatant was discarded, and the cell cultures were immediately washed three times with ice-cold PBS. The cells were fixed with 4% paraformaldehyde for the detection of fluorescence, in a routine manner. The fluorescence value of adriamycin was detected using a Synergy HTX multi-mode reader (BioTek Instruments, Winooski, VT, USA), and the excitation and emission wavelengths of adriamycin were 488 and 535 nm, respectively.

### 2.8. Cell Apoptosis Assay and Cell Cycle Assay

Cell apoptosis was detected by FCM. In short, cells were seeded into 6-well plates for 24 h and then subjected to different treatments. Cells were washed in cold PBS twice, stained with the binding buffer mixed with Annexin V- fluorescein isothiocyanate (FITC) and propidium iodide (PI) for 15 min in the dark, and then detected by FCM. The early and late apoptosis rates were analyzed by Flow Jo software.

For cell cycle assay, the cells were collected and placed in 75% cold ethanol for 1 h at −20 °C after 48 h of incubation with rutaecarpine. The ethanol was then discarded and cleaned with cold PBS. Cells were treated with RNase A (10 μg/mL) for 30 min at 37 °C to digest RNA, then stained with PI (50 μg/mL) for 15 min. Finally, cells were measured by FCM with an excitation wavelength of 480 nm through an FL-2 filter (585 nm). Mod Fit LT software was used to analyze the data.

### 2.9. Wound Healing and Cell Invasion Assay

Wound healing assay was conducted as previously described [41]. In brief, a single scratch wound was created using a sterile 10-μL plastic pipette tip across the cell surface. The area of a defined region within the scratch was measured using Image J software. The extent to which the wound had closed over 48 h was calculated and expressed as a percentage of the difference between time points 0 and 48 h.

Cell invasion assay was performed as previously described [42]. Briefly, the 8.0-μm pore size Trans-well inserts (FALCON, Corning, New York, NY, USA) and the 24-well plate were washed with PBS for 2 min before the experiment. The inserts were coated with 200 μL of Matrigel (dilution at 1:2; BD Biosciences). Cells resuspended in 0.2 mL serum-free medium at a density of 1 × 10^5^ cells/mL were transferred to the upper Matrigel chambers with 0.5 mL of 10% serum complete medium as the chemoattractant in the lower chamber and incubated at 37 °C for 48 h. Cells that were able to pass through the filter were fixed with 4% paraformaldehyde for 15 min and stained with 0.5% crystal violet for 30 min. Finally, the numbers of invaded cells in three randomly selected high-power fields were counted under the microscope.

### 2.10. Immunoprecipitation and Immunoblot Analysis

As described previously [43], cells were lysed by incubation for 15 min at 4 °C with 750 μL of lysis buffer (including 50 mM Tris-HCl, pH 8.0; 150 mM NaCl; 1% NP-40; 0.5% sodium deoxycholate; and 0.2% SDS) containing protease inhibitors. The lysates were then centrifuged at 13,000 × rpm for 15 min to obtain supernatants without cell debris and nuclei. After determining protein concentration, equal amounts of lysates were used for immunoprecipitation. The lysates were immunoprecipitated overnight at 4 °C with 2 μL indicated antibody and 25 μL protein A-Sepharose (Santa Cruz). Subsequently, the precipitates were washed three times (3000 × rpm, 1 min) with washing buffer, and the immune complexes were eluted with sample buffer containing 1% SDS for 10 min at 100 °C and analyzed by SDS-PAGE. Immunoblotting was performed using HRP-conjugated secondary antibodies and visualization with chemiluminescence as previously described.

### 2.11. Ubiquitination Assay and Cycloheximide Chase Assay

Cells were lysed with lysis buffer containing 1% SDS and then boiled at 100 °C for 10 min. Immunoprecipitation with appropriate antibodies was conducted as described previously [43]. Cells were transfected with the required plasmids and grown for 24 h. We added cycloheximide (CHX) at a final concentration of 100 μg/mL to inhibit protein synthesis. Equal numbers of cells were collected at the desired time points (0, 6, 12 h). The protein level of ABCB1 in the samples was determined by immunoblotting as described.

### 2.12. Construction of sh-MARCH8 Interference Plasmid

The MARCH8 interference (shMARCH8) sequences were as follows: forward, 5′-CCGGCCACTAACAGAGCCCAACTTTCTCGAGAAAGTTGGGCTCTGTTAGTGGTTTTT-3′; reverse, 5′-AATTAAAAACCACTAACAGAGCCCAACTTTCTCGAGAAAGTTGGGCTCTGTTAGTGG-3′. For insertion into the pLko.1-hygro vector, Age I and EcoRI restriction enzyme sites were fused to each terminus. The plasmid containing the MARCH8 interference sequence was confirmed by enzyme digestion and sequencing. MCF-7/ADR cells were transfected with the above constructs together with packaging lentivirus plasmids. After two weeks of selection with hygromycin, protein levels were tested by Western blotting. 

### 2.13. In Vivo Tumorigenesis Assay

Animal study was performed as previously described [44,45]. The BACB/C nude mice xenograft models were used for the in vivo study. Briefly, 2.5 × 10^6^ cells suspended in 100 μL ice-cold PBS were subcutaneously injected into the right flank of mice (female, 6 weeks old). When the tumors reached a mean volume of 35 mm^3^, mice were randomly divided into four groups (three mice in each experimental group) and received various treatments: (a) PBS, intraperitoneally (i.p.) every 2 days (q2d); (b) rutaecarpine alone (15 mg/kg, i.p. q2d); (c) adriamycin (15 mg/kg, i.p. q2d) or paclitaxel alone (15 mg/kg, i.p. q2d); (d) rutaecarpine (15 mg/kg, i.p. q2d) plus adriamycin (15 mg/kg, i.p. q2d) or paclitaxel (15 mg/kg, i.p. q2d). MCF-7 and MCF-7/ADR xenograft tumors were treated with adriamycin, while A549 and A549/Taxol used paclitaxel. Each animal was tagged on the buttocks with a different color and followed individually throughout the experiments. We monitored tumor growth starting on the first day of treatment and measured the volume of the xenograft every 5 days. Tumor volume (in cubic millimeters) was measured with calipers and calculated as (W^2^ × L)/2, where *W* is the width and *L* is the length. All animal experiments were approved by the institutional animal care and use committee of Chongqing University. The approval code: 2020035, approved on 3 September 2020.

### 2.14. Immunohistochemistry Assay

Tumors obtained from MCF-7, MCF-7/ADR, A549 and A549/Taxol xenograft mice were fixed in 4% paraformaldehyde, then paraffin-embedded and sectioned. Tumor slides were incubated with primary antibodies at 4 °C overnight, and then incubated with secondary antibody. The chromogenic reaction was conducted with 3,3-diaminobenzidine (DAB) and then we counterstained the nucleus with hematoxylin. Images were captured using Nikon microscope.

### 2.15. Statistical Analysis

The data were presented as the means and standard deviations (SD) from at least three independent experiments. Data were analyzed with GraphPad Prism software. A *p*-value of <0.05 was considered statistically significant.

## 3. Results

### 3.1. Rutaecarpine Significantly Enhances the Sensitivity of ABCB1-Overexpressing Cancer Cells to Anticancer Drugs

The structure of rutaecarpine is listed in Supplementary Figure S1. Firstly, the cell counting kit-8 (CCK8) assay was used to evaluate the toxicity of rutaecarpine in the ABCB1-overexpressing and corresponding parental cell lines. Based on the toxicity results, we could choose concentrations that would not significantly influence cell viability. Hence, 5 and 20 μM of rutaecarpine were selected to conduct further experiments (Supplementary Figure S1). In addition, the Annexin V-FITC/PI double staining assay and PI staining assay were used to determine cell apoptosis and cell cycle, respectively. According to the results, rutaecarpine at a concentration of 20 μM had little effect on cell apoptosis and the cell cycle of cancer cells (Supplementary Figure S2). Therefore, rutaecarpine at 20 μM was identified as a non-toxic concentration to cells.

**Figure 1:**
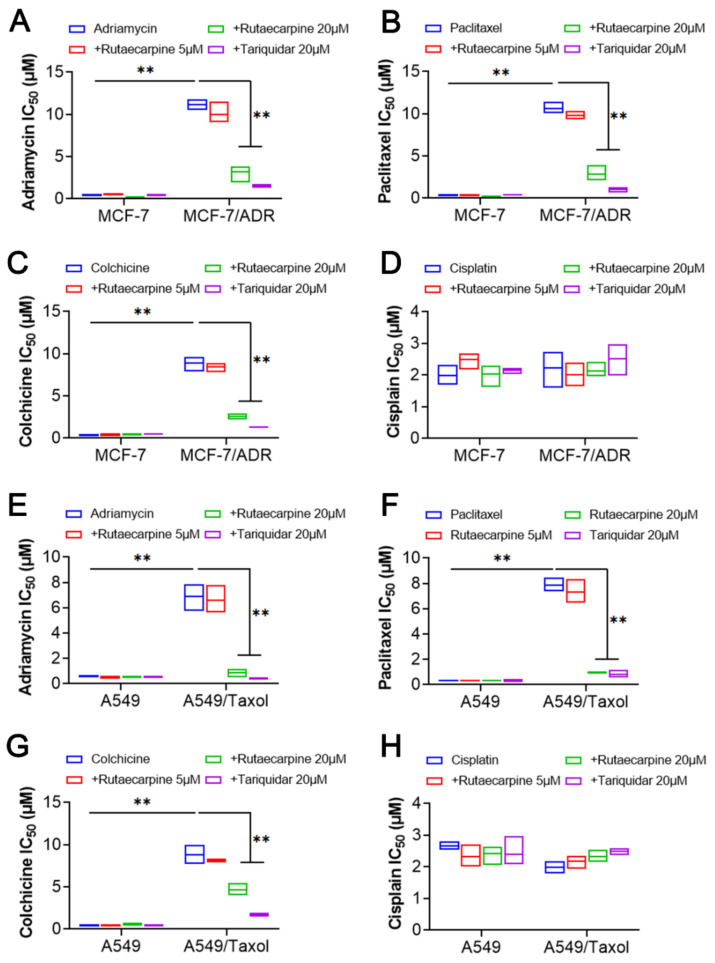
The effects of rutaecarpine on the IC_50_ values of different anticancer drugs in parental and ABCB1-overexpressing cancer cells. IC_50_ values of (**A**) adriamycin, (**B**) paclitaxel, (**C)** colchicine, and (**D**) cisplatin in parental MCF-7 and drug-resistant ABCB1-overexpressing MCF-7/ADR cells. IC_50_ values of (**E**) adriamycin, (**F**) paclitaxel, (**G**) colchicine, and (**H**) cisplatin in parental A549 and drug-resistant ABCB1-overexpressing A549/Taxol cells with or without treatment of rutaecarpine. All data are presented from three independent experiments. ** *p* < 0.01.

To investigate the reversal effects of rutaecarpine on ABCB1-overexpressing cells, cell survival assays were performed by CCK8 in the presence or absence of rutaecarpine. In this study, adriamycin, paclitaxel, and colchicine were used as ABCB1 transport substrates. Cisplatin, a non-ABCB1 transport substrate drug, was selected as a negative control. In addition, tariquidar at 20 μM was used as a positive control inhibitor of ABCB1. As shown in Figure 1, rutaecarpine significantly sensitized ABCB1-overexpressing cancer cells MCF-7/ADR (Figure 1A–C) and A549/Taxol (Figure 1E–G) to ABCB1 transport substrates, compared with the resistance cells without rutaecarpine, and this sensitization occurred in a dose-dependent manner. However, rutaecarpine showed no significant difference in its cytotoxic effect in the parental cell lines MCF-7 and A549. Furthermore, when combined with cisplatin, rutaecarpine showed no significant difference in its cytotoxic effect in either the resistant cell lines or the parental cell lines (Figure 1D,H). Collectively, these results show that rutaecarpine significantly reverses the drug resistance of ABCB1-overexpressing cells to adriamycin, paclitaxel, and colchicine.

### 3.2. Rutaecarpine Downregulates the Protein Expression Level of ABCB1

**Figure 2:**
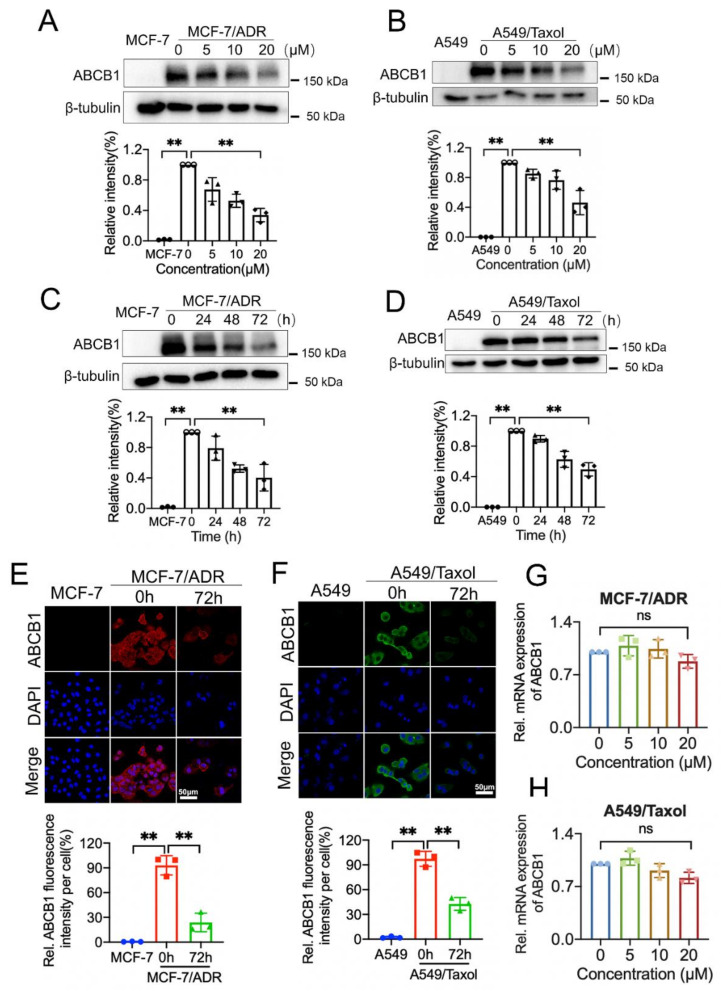
Rutaecarpine could alter the protein expression and subcellular localization of ABCB1 transporter. (**A**) ABCB1 expression in parent MCF-7 or MCF-7/ADR cells incubated with 0, 5, 10, 20 μM of rutaecarpine for 48 h was detected by Western blot and the relative intensity is shown. (**B**) ABCB1 expression in parent A549 or A549/Taxol cells treated with 0, 5, 10, 20 μM of rutaecarpine for 48 h was analyzed as in (**A**) and the relative intensity is shown. (**C**) ABCB1 expression in MCF-7 or MCF-7/ADR cells incubated with 20 μM of rutaecarpine for 0, 24, 48, and 72 h was detected and the relative intensity is shown. (**D**) Detection and relative intensity of ABCB1 expression in A549 or A549/Taxol cells incubated with 20 μM of rutaecarpine for 0, 24, 48, and 72 h are shown. (**E**) Subcellular localization of ABCB1 expression in MCF-7/ADR cells incubated with 20 μM of rutaecarpine for 72 h. (**F**) Subcellular localization of ABCB1 expression in A549/Taxol cells incubated with 20 μM of rutaecarpine for 72 h. Red: ABCB1 in MCF-7/ADR cells. Green: ABCB1 in A549/Taxol cells. Blue: DAPI counterstains the nuclei. MCF-7 and A549 represented the negative control group. (**G**,**H**) qRT-PCR was used to detect the expression levels of ABCB1 in MCF-7/ADR and A549/Taxol cells after 48 h of treatment with various concentrations of rutaecarpine; β-actin was used as an internal control. All data are presented as mean ± SD of three independent experiments. ns, not significant. ** *p* < 0.01.

To find out the underlying mechanism of sensitivity enhancement to apoptosis by rutaecarpine, we tested whether rutaecarpine plays a role by inhibiting the efflux activity, downregulating protein expression, or changing the subcellular localization of the transporter. We carried out Western blotting and immunofluorescence (IFC) assays in ABCB1-overexpressing cells (MCF-7/ADR and A549/Taxol) and their parental cells (MCF-7 and A549) to investigate these possibilities. As shown in Figure 2A,B, treatment with 20 μM of rutaecarpine for 48 h had a significant effect on the expression level of the ABCB1 protein in ABCB1-overexpressing cells. Then, cells were incubated with 20 μM of rutaecarpine for a different time period. As shown in Figure 2C,D, rutaecarpine significantly downregulated the protein expression level of ABCB1 in a time-dependent manner. Moreover, immunofluorescence staining showed that ABCB1 expression was significantly reduced after the treatment of rutaecarpine (Figure 2E,F), which was consistent with Western blotting results. Finally, qRT-PCR was used to detect the effect of rutaecarpine on the mRNA level of ABCB1, and we found that rutaecarpine did not significantly downregulate its mRNA level compared with the control group (Figure 2G,H). These data indicated that the reversal of MDR by rutaecarpine was caused by the decreased expression of the ABCB1 protein.

### 3.3. The Decrease in ABCB1 Promotes Intracellular Drug Accumulation

**Figure 3:**
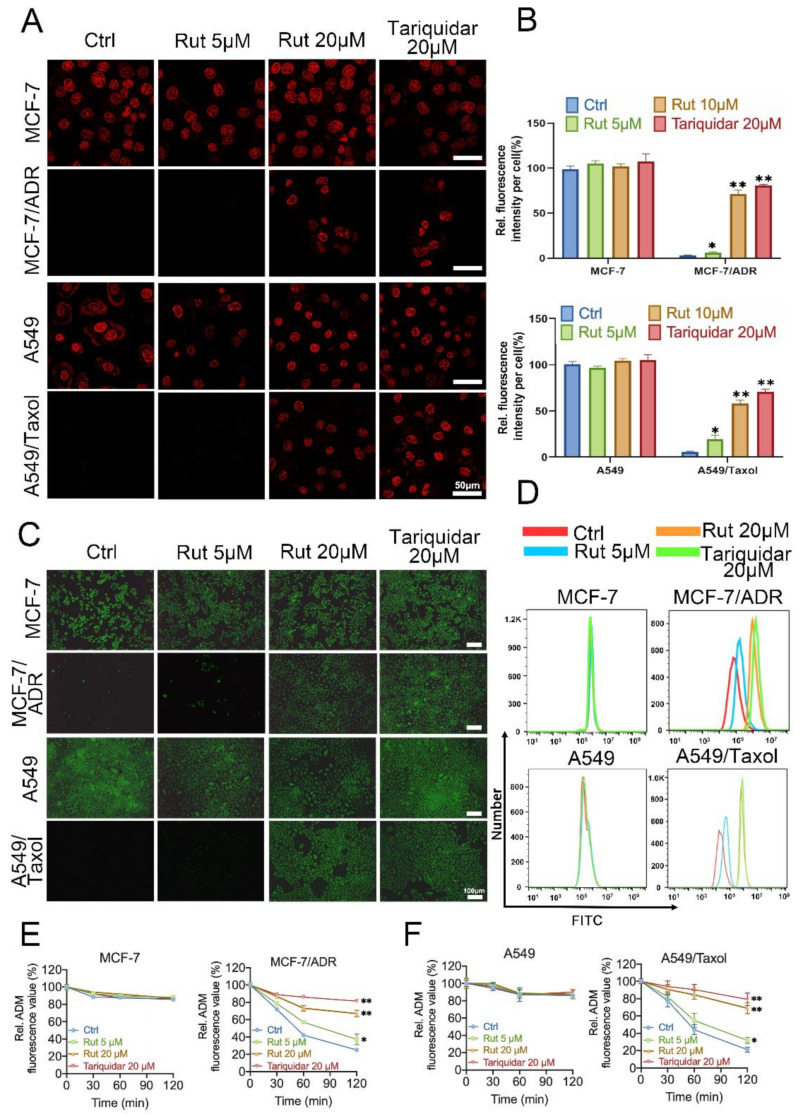
Effects of rutaecarpine on accumulation and efflux in ABCB1-overexpressing cancer cells. (**A**) Cells were observed by laser scanning confocal microscope (LSCM), and the representative images and (**B**) quantitative analysis of adriamycin fluorescence are shown. Adriamycin staining is shown in red. Data are presented as mean ± SD of three independent experiments. (**C**) Cells were observed by fluorescence microscopy, and the representative images of the accumulation of rhodamine 123 are shown. (**D**) Cells were detected by FCM, and the quantitative analysis of rhodamine 123 fluorescence is shown. (**E**,**F**) The effects of rutaecarpine on efflux of adriamycin in MCF-7, MCF-7/ADR, A549, and A549/Taxol cells. Tariquidar (20 μM) was used as a positive control inhibitor of ABCB1. Data are represented as mean ± SD of three independent experiments. * *p* < 0.05 and ** *p* < 0.01.

The above results demonstrated that rutaecarpine could antagonize ABCB1-mediated MDR by altering the protein expression level of ABCB1. In order to further explore whether the alteration of ABCB1 protein levels will affect the intracellular drug concentration, intracellular accumulations of rhodamine 123 and adriamycin were detected in the ABCB1-overexpressing cells and corresponding parental cells treated with rutaecarpine or not. As shown in Figure 3A–C, rutaecarpine dramatically increased the intracellular level of adriamycin and rhodamine 123 in ABCB1-overexpressing MCF-7/ADR and A549/Taxol cells in a dose-dependent manner, while no significant change was found in parental MCF-7 and A549 cells after treatment with 20 μM of rutaecarpine for 48 h. Subsequently, FCM analysis was used to detect the intracellular level of rhodamine 123, which is consistent with the previous results (Figure 3D). These results indicated that the downregulation of the ABCB1 protein level leads to the accumulation of drugs. 

Next, we performed efflux assays with drug-resistant cells and corresponding parental cells to investigate whether the intracellular drug efflux was influenced by the downregulation of ABCB1. As shown in Figure 3E,F, the efflux efficiency of adriamycin in MCF-7/ADR and A549/Taxol cells treated with rutaecarpine for 48 h was lower than that in untreated cells. However, rutaecarpine could not significantly affect the efflux of adriamycin in the parental MCF-7 and A549 cells. Collectively, these results suggested that the decrease in ABCB1 protein levels caused by prolonged pretreatment with rutaecarpine was in a position to slow down the rate of drug efflux to facilitate drug accumulation.

### 3.4. Rutaecarpine Enhances Adriamycin/Paclitaxel-Induced Apoptosis in ABCB1-Overexpressing Cells

**Figure 4:**
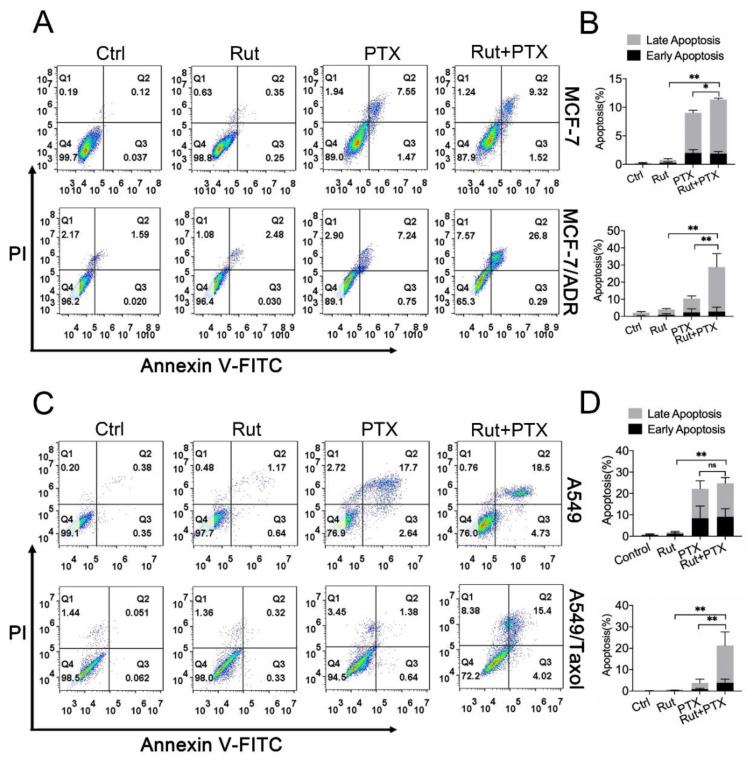
The effects of rutaecarpine combined with anticancer drugs on the apoptosis of parental and ABCB1-overexpressing cells. (**A**–**D**) The apoptosis was detected by FCM with Annexin V/PI staining and the percentage of apoptotic cells was analyzed. Various treatments were given as follows: MCF-7 and A549 cells were treated with 20 μM rutaecarpine, 500 nM paclitaxel alone or in combination for 48 h. MCF-7/ADR and A549/Taxol cells were treated with 20 μM rutaecarpine and 5 μM paclitaxel alone or in combination for 48 h. Rut, rutaecarpine; ADM, adriamycin; PTX, paclitaxel. Data are presented as mean ± SD of three independent experiments, * *p* < 0.05 and ** *p* < 0.01.

To further examine the effect of rutaecarpine in combination with adriamycin or paclitaxel on the apoptosis of cancer cells, cells were incubated under different conditions for 48 h. As shown in Supplementary Figure S3, the combination of rutaecarpine with adriamycin or paclitaxel significantly increased the number of apoptotic cells in drug-resistant cells. To quantify the number of apoptotic cells, we measured the apoptosis rate by FCM. In this experiment, we replaced the adriamycin that induces apoptosis in MCF-7 and MCF-7/ADR cells with paclitaxel as adriamycin has red fluorescence, which coincides with the PI channel of the reagent for detecting apoptosis (previous results showed that the effect of paclitaxel on MCF-7 and MCF-7/ADR cells is similar to that of adriamycin). Compared with the treatment of paclitaxel alone, the co-administration of rutaecarpine and paclitaxel vastly increased the apoptosis rate in MCF-7/ADR and A549/Taxol cells, but had less effect on MCF-7 and A549 cells (Figure 4C,D). These results suggested that rutaecarpine could enhance adriamycin/paclitaxel-induced apoptosis in ABCB1-overexpressing cancer cells.

### 3.5. Rutaecarpine Combines with Anticancer Drugs to Inhibit the Migration and Invasion of ABCB1-Overexpressing Cells

**Figure 5:**
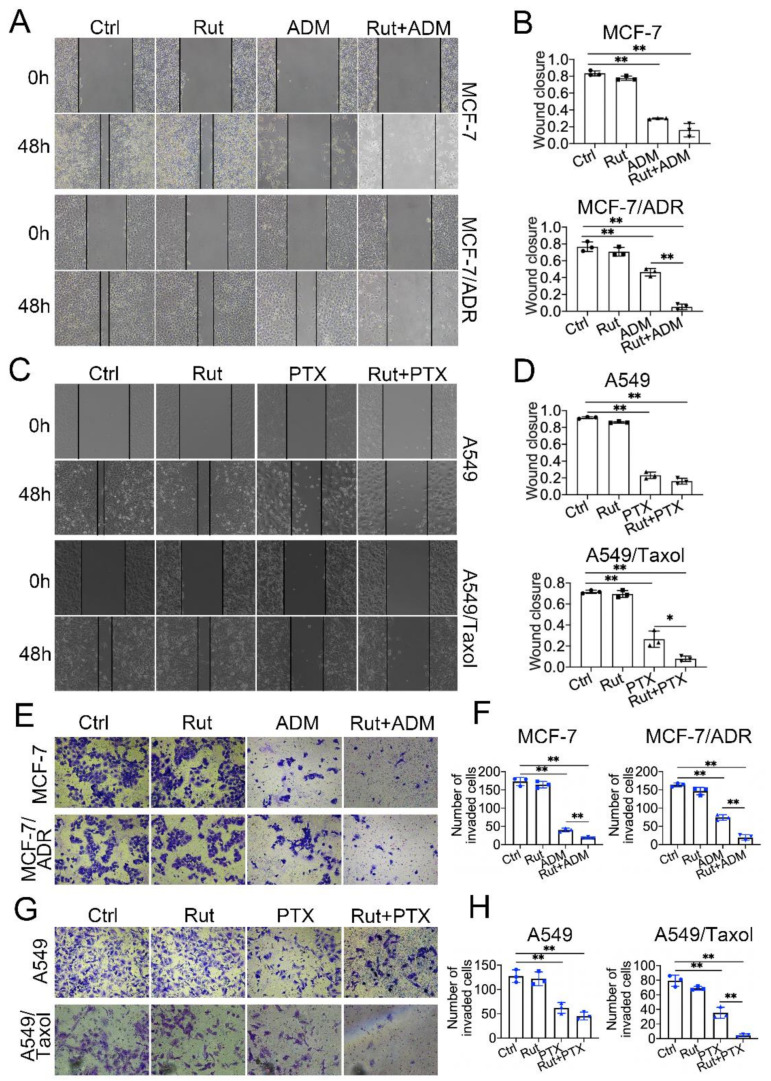
The effects of rutaecarpine combined with anticancer drugs on the mobility of parental and ABCB1-overexpressing cells. (**A**) Wound healing assay of MCF-7 and MCF-7/ADR cells following treatment with 20 μM rutaecarpine and 500 nM or 5 μM adriamycin alone or in combination, and (**B**) the quantification of the wound closure area. Magnification, ×100. Data are mean ± SD (*n* = 3). (**C**) Wound healing assay of A549 and A549/Taxol cells following treatment with 20 μM rutaecarpine and 500 nM or 5 μM paclitaxel alone or in combination, and (**D**) the quantification of the wound closure area. Magnification, ×100. Data are mean ± SD (*n* = 3). (**E**) Migration and invasion assays of MCF-7 and MCF-7/ADR cells following treatment with 20 μM rutaecarpine and 500 nM or 5 μM adriamycin alone or in combination for 48 h, and (**F**) the results of the migration and invasion assays were statistically analyzed. Data are mean ± SD (*n* = 3). (**G**) Migration and invasion assays of A549 and A549/Taxol cells following treatment with 20 μM rutaecarpine and 500 nM or 5 μM paclitaxel alone or in combination for 48 h, and (**H**) the results of the migration and invasion assays were statistically analyzed. Magnification, ×100. Data are mean ± SD (*n* = 3). * *p* < 0.05 and ** *p* < 0.01. Rut, rutaecarpine; ADM, adriamycin; PTX, paclitaxel.

To evaluate whether combination treatment affects tumor migration and metastasis, wound healing and trans-well assays were conducted in MCF-7/ADR and A549/Taxol cells together with their corresponding controls. The wound healing assays revealed that the migratory ability of MCF-7/ADR and A549/Taxol cells was significantly reduced with the combination treatment for 48 h compared with the controls, while no significant difference was found in MCF-7 and A549 cells (Figure 5A–D). The cell invasion assays showed that the number of invading cells of MCF-7/ADR and A549/Taxol was significantly lower in the co-treatment group than that in other groups (Figure 5E–H). These results suggested that combined treatment has a better effect in blocking the cell migration and metastasis of ABCB1-overexpressing cells.

### 3.6. RNA-Seq Indicates MARCH8 Is Involved in Rutaecarpine-Induced ABCB1 Downregulation

**Figure 6:**
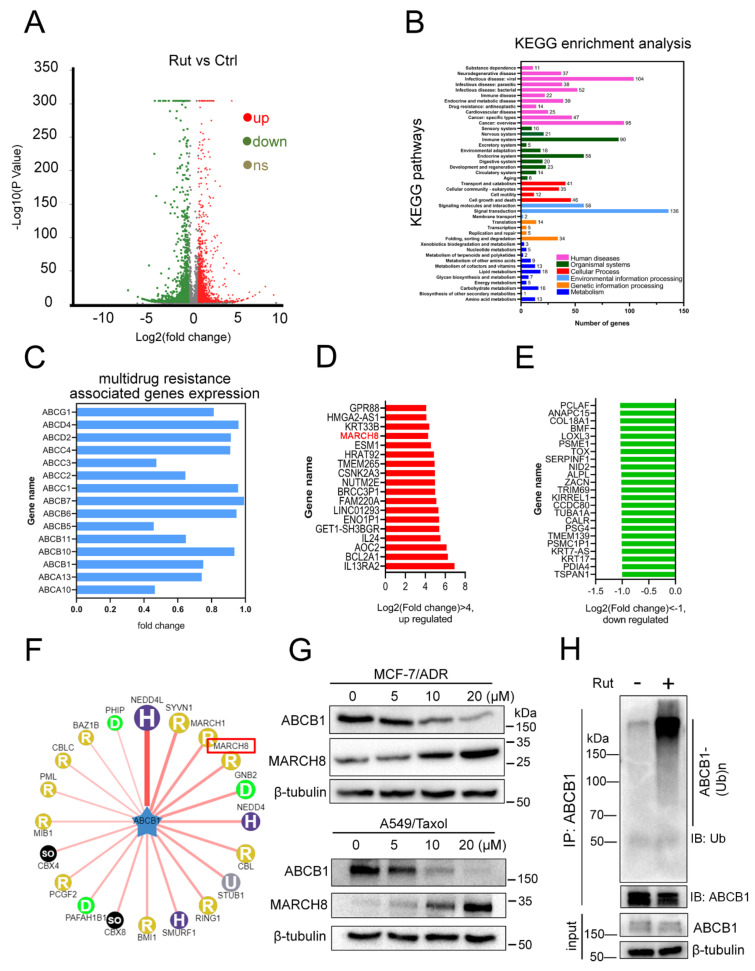
MCF-7/ADR cells treated with rutaecarpine were subjected to RNA-seq analysis. (**A**) A volcano plot shows the number of upregulated, downregulated, and non-differentially expressed genes. Green color represents fold change ≤ 0.5 and red color represents fold change ≥ 2 fold. (**B**) KEGG classification of DEGs in MCF-7/ADR cells treated with rutaecarpine. (**C**–**E**) Transcriptome analysis of genes differentially expressed between control and cells treated with rutaecarpine. Expression of multidrug resistance-associated genes is marked with blue (**C**), rutaecarpine-activated genes are marked with red (**D**), and rutaecarpine-repressed genes are marked with green (**E**). (**F**) The network view of the predicted E3 ligase of ABCB1 by UbiBrowser. (**G**) The expression levels of ABCB1 and MARCH8 in MCF-7/ADR and A549/Taxol cells treated with rutaecarpine (0–20 μM, 48 h) were detected by Western blotting. β-tubulin was used as the loading control. (**H**) The ubiquitination of endogenous ABCB1 was analyzed by immunoprecipitation with anti-ABCB1 antibody and Western blotting with anti-ubiquitin antibody in control or cells treated with rutaecarpine. β-tubulin was used as a loading control.

RNA-seq was used to gain insight into the molecular function of rutaecarpine on ABCB1-overexpressing cells. The steps including RNA isolation, cDNA library preparation, and RNA-seq were completed by Shanghai Majorbio Bio-pharm Technology Co., Ltd. (Shanghai, China). The dataset involved can be viewed in Table S1. The fold change (FC) of gene expression was calculated relative to the control cells, and genes with log2|FC| > 0.5 were considered as differentially expressed. The volcano plot showed that 1716 differentially expressed genes (DEGs) were upregulated and 1165 DEGs were downregulated, and 1425 DEGs were unchanged in RNA-seq data (Figure 6A). According to Kyoto Encyclopedia of Genes and Genomes (KEGG) enrichment analysis, these DEGs were involved in 43 pathways, mainly mediating signal transduction, human diseases, and metabolism (Figure 6B). Next, we compared the mRNA expression pattern between control and drug-treated cells (Figure 6C–E). As shown in Figure 6C, compared with the control group, the mRNA expression level of ABCB1 was slightly decreased, but there was no significant difference, which is consistent with our previous results (Figure 2G). These data suggested that the marked decrease in the ABCB1 protein was probably not caused by the alteration of the transcription level. Figure 6D includes 18 upregulated genes, and Figure 6E depicts 23 downregulated genes in the rutaecarpine treatment MCF-7/ADR cells. Interestingly, the E3 ubiquitin ligase MARCH8 was detected in the upregulated genes, suggesting that the downregulation of ABCB1 might be through the ubiquitin pathway. We then used the web tool UbiBrowser (http://ubibrowser.ncpsb.org/ubibrowser/home/index) [46] to predict the E3 ubiquitin ligase of ABCB1 and found that MARCH8 was a candidate (Figure 6F). Western blotting analysis verified that the protein expression level of MARCH8 was increased by rutaecarpine in a dose-dependent manner (Figure 6G). Finally, we tested the ubiquitination level of ABCB1 after rutaecarpine treatment. As we expected, rutaecarpine could significantly enhance the ubiquitination level of ABCB1 (Figure 6H). Therefore, these results demonstrated that the degradation of ABCB1 might be caused by the increase in its ubiquitination level through E3 ubiquitin ligase MARCH8.

### 3.7. E3 Ubiquitin Ligase MARCH8 Interacts with ABCB1 and Promotes Its Ubiquitination and Degradation

**Figure 7:**
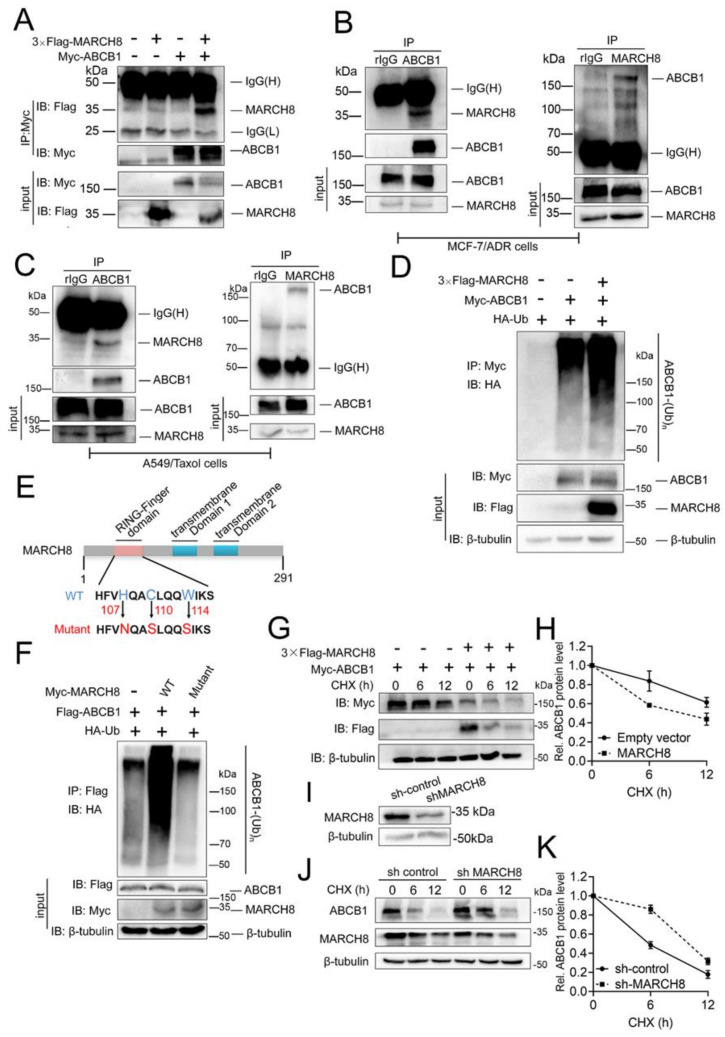
ABCB1 interacts with MARCH8 and positively regulates ABCB1 degradation. (**A**) The Myc-ABCB1 expression plasmid was co-transfected with or without 3 × Flag-MARCH8 into HEK293T cells. The ABCB1 protein was co-immunoprecipitated (Co-IP) with the anti-Myc antibody, and the expression of MARCH8 and ABCB1 in the whole-cell lysate (WCL) was confirmed by immunoblotting (IB) with antibodies against Flag and Myc. (**B**) The endogenous interaction of ABCB1 and MARCH8 was tested in MCF-7/ADR cells. (**C**) The endogenous interaction of ABCB1 and MARCH8 was tested in A549/Taxol cells. (**D**) HA-ubiquitin, 3 × Flag-MARCH8, and Myc-ABCB1 plasmids were co-transfected into HEK293T cells. ABCB1 ubiquitination was detected by Co-IP of ABCB1 with the anti-Myc antibody and Western blotting with anti-HA antibody. The protein expression levels of ABCB1 and MARCH8 in the whole-cell lysates were confirmed. MG132 was added during cell culture to inhibit the degradation of ABCB1. (**E**) Schematic representation of MARCH8 and its point mutants. (**F**) HA-ubiquitin and Flag-ABCB1 expression plasmids were co-transfected into HEK293T cells with Myc-MARCH8 or with MARCH8 mutants. The effects of MARCH8 and its mutants on ABCB1 ubiquitination were analyzed as for panel D. MG132 was added during cell culture to inhibit the degradation of ABCB1. (**G**,**H**) MARCH8 expression plasmids or empty vectors were co-transfected with Myc-ABCB1 plasmids into HEK293T cells. 36 hours later, the transfected cells were treated with 100 μg/mL cycloheximide (CHX) for the indicated time. The effects of ABCB1 protein stability were analyzed by Western blotting (**G**) and quantified (**H**). (**I**) The expression level of MARCH8 between control and knockdown was tested by Western blotting, and β-tubulin was used as a loading control. (**J**,**K**) ABCB1 protein stability was analyzed between control and MARCH8 knockdown in MCF-7/ADR cells as for panel G. The expression of ABCB1 and MARCH8 was determined by Western blotting (**J**) and quantified (**K**). β-tubulin was used as a loading control. Error bars represent mean ± SD of three independent experiments.

In previous experiments, we have shown that rutaecarpine could significantly downregulate the protein level of ABCB1, but has no significant effect on its mRNA level (Figure 2). Transcriptome analysis led us to speculate that the ABCB1 protein level was downregulated by MARCH8. Next, we performed co-immunoprecipitation (Co-IP) experiments in HEK293T cells to verify the interaction between ABCB1 and MARCH8. ABCB1 was shown to pull down MARCH8 (Figure 7A). We then demonstrated that endogenous ABCB1 and MARCH8 proteins also interact with one another in MCF-7/ADR and A549/Taxol cells (Figure 7B,C). 

As an E3 ubiquitin ligase, MARCH8 could use the ubiquitin–proteasome system (UPS) to degrade targeted proteins [47]. To evaluate whether MARCH8 could promote ABCB1 ubiquitination, we transfected MARCH8, ubiquitin (Ub), and ABCB1 plasmids into HEK293T cells, and 48 h later, polyubiquitinated ABCB1 proteins were detected using an anti-hemagglutinin (anti-HA) antibody (Figure 7D). Wild-type (WT) MARCH8 dramatically enhanced ABCB1 ubiquitination, while the catalytically inactive mutant (Figure 7E) had no effect (Figure 7F). Therefore, MARCH8 is an E3 ubiquitin ligase for ABCB1 and ABCB1 ubiquitination depended on the E3 ubiquitin ligase catalytic activity of MARCH8. 

Ubiquitination might promote protein degradation through UPS machinery [48]. To determine whether MARCH8 could promote ubiquitination-mediated ABCB1 degradation, we transfected MARCH8 and ABCB1 plasmids into HEK293T cells and measured the ABCB1 protein level. As expected, MARCH8 significantly shortened the half-life of the ABCB1 protein (Figure 7G,H). To further test whether endogenous MARCH8 could promote ABCB1 protein degradation, we knocked down MARCH8 using short hairpin RNA (shRNA) in MCF-7/ADR cells (Figure 7I) and measured the ABCB1 protein half-lives with the cycloheximide (CHX) chase assay. The half-life of the ABCB1 protein was prolonged after MARCH8 depletion (Figure 7J,K). Collectively, these results suggested that MARCH8 is an E3 ubiquitin ligase for ABCB1 and negatively regulates ABCB1 protein stability by regulating its ubiquitination.

Besides that, in order to verify whether rutaecarpine downregulates the ABCB1 protein via MARCH8, a reversal experiment was performed in MARCH8 knockdown MCF-7/ADR cells. As shown in Supplementary Figure S4, there was no significant reduction in ABCB1 in MARCH8 knockdown MCF-7/ADR cells after treatment with various concentrations of rutaecarpine. Similarly, the deletion of MARCH8 also greatly reduced the sensitivity of drug-resistant cell lines to adriamycin. These results suggested that the knockdown of MARCH8 partially prevented the reversal effect of rutaecarpine on ABCB1. Based on these analyses, we propose that rutaecarpine is most likely to regulate ABCB1 through MARCH8.

### 3.8. Combined Treatment of Rutaecarpine and Anticancer Drugs Inhibits the Growth of Xenograft Tumors

**Figure 8:**
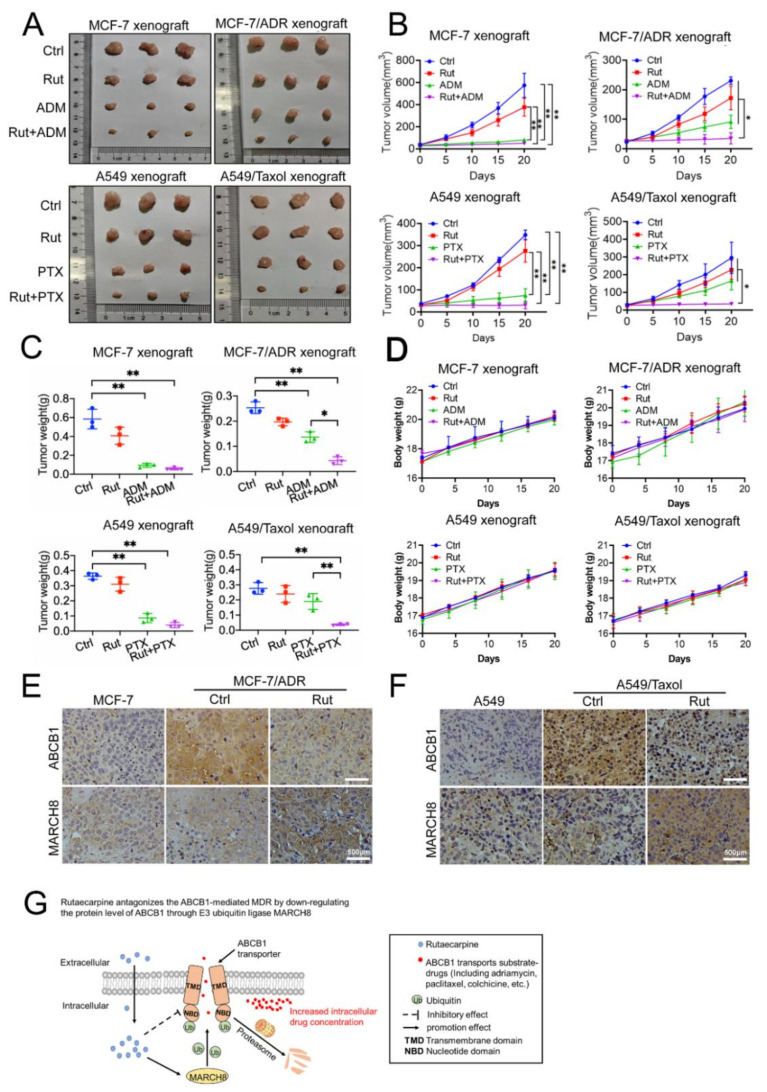
Potentiation of the antitumor effects of adriamycin/paclitaxel by rutaecarpine in the xenograft mice injected with parental or drug-resistant cells. (**A**) Tumor size, the photograph of xenografts was taken on the 20th day after implantation. Ctrl, control; Rut, rutaecarpine; ADM, adriamycin; PTX, paclitaxel. (**B**) Changes in tumor volume with time after different cells’ inoculation. Data shown are means ± SD for each group of three mice (* *p* < 0.05, ** *p* < 0.01). (**C**) The average tumor weight of each group was calculated after the tumors were resected from mice. Various treatments were given as follows: PBS (q2d); adriamycin/paclitaxel (15 mg/kg, i.p. q2d); rutaecarpine (15 mg/kg, i.p. q2d); rutaecarpine (15 mg/kg, i.p. q2d) and adriamycin (15 mg/kg, i.p. q2d)/paclitaxel (15 mg/kg, i.p. q2d). Data shown are means  ±  SD for each group of three mice (* *p* < 0.05, ** *p* < 0.01). (**D**) Changes in body weight after cell inoculation. Data shown are means ± SD for each group of three mice after implantation. (**E**,**F**) The expressions of ABCB1 and MARCH8 were detected by immunohistochemical staining (scale bar, 500 μm). (**G**) The working model of rutaecarpine.

The MCF-7, MCF-7/ADR, A549, and A549/Taxol xenograft models were established in BACB/C nude mice to evaluate the anti-tumor ability of the combination of rutaecarpine and anticancer drugs. After 20 days, tumor weight and size in the combination treatment group were significantly smaller compared with the control group. Especially in MCF-7/ADR and A549/Taxol xenograft tumors, the combined treatment group had much better therapeutic effect than other single drug treatments (Figure 8A–C). Moreover, no significant body weight loss or mortality was observed, indicating that the combination regimen was tolerated by the nude mice (Figure 8D). In addition, immunohistochemical (IHC) staining also demonstrated that the expression level of ABCB1 was downregulated, while MARCH8 was upregulated (Figure 8E,F). As shown in the model, these results implied that rutaecarpine might reduce the expression level of ABCB1 by upregulating MARCH8 expression, thus suggesting a therapeutic potential via combination use with other anticancer drugs for the treatment of multidrug-resistant tumors (Figure 8G).

## 4. Discussion

It is generally known that the overexpression of ABCB1 could induce MDR in some but not all types of cancer cells [7,49]. Despite the great progress in cancer treatment, MDR is still a main obstacle to the success of cancer chemotherapy. Growing evidence showed that the increased risk of adverse events during treatment in some types of cancers, including acute myeloid leukemia (AML), ovarian cancer, osteosarcomas, and melanoma, is closely related to the high expression of ABCB1 [19,50,51,52,53,54]. Therefore, the combination of ABCB1 inhibitors and anticancer drugs is identified as a promising approach to overcome ABCB1-mediated MDR. It is urgent to develop efficient and safe reversal agents to inhibit MDR by either decreasing the expression level of ABCB1 proteins or inhibiting the efflux function of ABCB1. However, a series of clinical trials targeting ABCB1 to overcome MDR have failed due to sub-optimal efficacy and adverse effects. So far, no ABCB1 inhibitor has been approved by the FDA for clinical treatment [55,56,57]. Many recent studies demonstrated that combinations of chemotherapeutic drugs and a kinase inhibitor (KI) have the capacity to reverse ABCB1-mediated MDR [22,39,58]. However, there are few studies evaluating whether alkaloids extracted from traditional Chinese medicine have the potential to reverse ABCB1-mediated MDR.

Rutaecarpine is the major alkaloid component isolated from *Evodia rutaecarpa*, a traditional Chinese herbal medicine. Rutaecarpine has long been explored for the potential treatment of cardiovascular diseases, gastrointestinal disorders, headaches, Alzheimer’s disease, and acute pancreatitis [59,60,61,62]. Previous studies have indicated that rutaecarpine performs multiple anti-atherosclerotic functions, including anti-thrombotic, anti-inflammatory, and anti-obesity activity [63,64]. Here, we reported for the first time that rutaecarpine shows a significant effect on ABCB1-mediated MDR at a non-toxic concentration. 

In this study, we found that rutaecarpine, at a non-toxic concentration, significantly sensitized ABCB1-overexpressing cancer cells to their substrates, respectively, in a dose-dependent manner. Firstly, we carried out CCK8 assays to test the toxicity of rutaecarpine and obtain the relatively non-toxic concentration for the reversal study. Based on these results, 5 and 20 μM of rutaecarpine were selected for the reversal studies. Our data indicated that rutaecarpine significantly increased the efficacy of adriamycin, paclitaxel, and colchicine to the ABCB1-overexpressing MCF-7/ADR and A549/Taxol cells compared to untreated control resistant cells in a dose-dependent manner. The reversal effect of rutaecarpine was lower than that of ABCB1 inhibitor tariquidar. Furthermore, rutaecarpine had no effect on the toxicity of non-substrate drug cisplatin. These results indicate that rutaecarpine exclusively reversed ABCB1-mediated MDR.

The reversal of ABCB1-mediated MDR may be related to the downregulation of ABCB1 protein expression and/or the alteration of subcellular localization. Our results showed that rutaecarpine could change the protein level of ABCB1 in a dose/time-dependent manner. Subsequently, we verified the effect of rutaecarpine on the mRNA level of ABCB1. Although it has a slight effect on its mRNA, it is not the main reason for the decrease in ABCB1 protein expression. Thus, rutaecarpine exerts a reversal effect by downregulating the protein level of ABCB1. Then, drug accumulation and efflux experiments were conducted to determine whether the downregulation of the ABCB1 protein level would affect its drug efflux function. We found that prolonged rutaecarpine treatment significantly increased the intracellular accumulation of drugs in resistant cells, probably resulting from the decrease in the ABCB1 transporter. Furthermore, the data showed no significant difference in drug accumulation and efflux rate in parental cells. Finally, we used flow cytometry, wound healing, cell invasion, and other experiments to evaluate the therapeutic effect of rutaecarpine combined with anticancer drugs. Moreover, we evaluated the feasibility and toxicity of the combination strategy through animal experiments. Compared with other treatment groups, the tumor growth in the combined treatment group was significantly slowed and no significant body weight loss or mortality was observed, indicating that the combination regimen was tolerated by the nude mice. These results showed that rutaecarpine can antagonize ABCB1-mediated MDR in a safe and effective way. 

To further explore the mechanism of rutaecarpine downregulating the ABCB1 protein level, we analyzed RNA-seq data obtained from MCF-7/ADR cells treated with rutaecarpine. Consistent with the previous results, rutaecarpine did not significantly downregulate the ABCB1 transcription level. Based on previous results, we speculated whether rutaecarpine promoted ABCB1 to undergo post-translational modification to increase its degradation. Since protein degradation is mostly related to the ubiquitination pathway, we selected MARCH8 from transcriptome analysis and E3 ubiquitin ligase candidates predicted by web tool UbiBrowser for verification. Western blotting analysis showed that the protein level of MARCH8 indeed increased significantly after treatment with rutaecarpine. Then, we carried out protein–protein interaction experiments. As we expected, ABCB1 is a newly discovered substrate of MARCH8. MARCH8 can interact with ABCB1 and promote its ubiquitination and degradation. We also proved that the ubiquitination of ABCB1 by MARCH8 relies on the catalytic activity of its RING finger domain. In order to determine whether rutaecarpine plays a role through MARCH8, we used the MARCH8 knockdown MCF-7/ADR cells for further verification. With the knockdown of MARCH8, treatment with different concentrations of rutaecarpine did not cause a significant decrease in the ABCB1 protein. In addition, the reversal effect of rutaecarpine on drug-resistant cells was partially damaged. Based on the obtained results, we suggested that MARCH8 is essential for the reversal function of rutaecarpine.

In conclusion, our study demonstrated the great potential of rutaecarpine in the reversal of tumor drug resistance. Different from many previous studies, rutaecarpine reverses ABCB1-mediated MDR by downregulating the protein level of ABCB1, rather than directly inhibiting the drug efflux activity of ABCB1. Thus, the therapeutic strategy of using rutaecarpine in combination with other anticancer drugs may be a novel method for cancer treatment in the future.

## Data Availability

All data generated or analyzed during this study are included in this published article and its Supplementary Materials.

## Supplementary Materials

The following are available online at https://www.mdpi.com/article/10.3390/biomedicines9091143/s1, Figure S1: Chemical structure and cytotoxicity of rutaecarpine in parental and ABCB1-overexpressing cells, Figure S2: The effect of rutaecarpine on cells at 20 Μm, Figure S3: The effects of rutaecarpine combined with anticancer drugs on the apoptosis of parental and ABCB1-overexpressing cells, Figure S4: Knockdown of MARCH8 partially prevented the reversal effect of Rutaecarpine on ABCB1, Table S1: Total genes after rutaecarpine treatment in MCF-7/ADR cells was shown.
