# Supplementary material for: Rutaecarpine Increases Anticancer Drug Sensitivity in Drug-Resistant Cells through MARCH8-Dependent ABCB1 Degradation
Source: Biomedicines. 2021 Sep 2;9(9):1143. doi: 10.3390/biomedicines9091143 (PMC8466742; doi:10.3390/biomedicines9091143)
Supplement: Supplementary file 1 [file biomedicines-09-01143-s001.zip › supplementary figures.pdf]

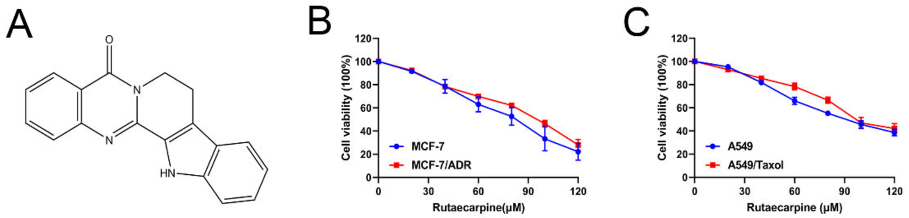

Figure S1. Chemical structure and cytotoxicity of rutaecarpine in parental and ABCB1-overexpressing cells. **(A)** Chemical structure of rutaecarpine. **(B)** Concentration-viability curves for MCF-7 and MCF-7/ADR cells incubated with rutaecarpine for 48 hours. **(C)** Concentration-viability curves for A549 and A549/Taxol cells incubated with rutaecarpine for 48 hours. Error bars represent as mean ± SD of three independent experiments (mean ± SD of n = 3)

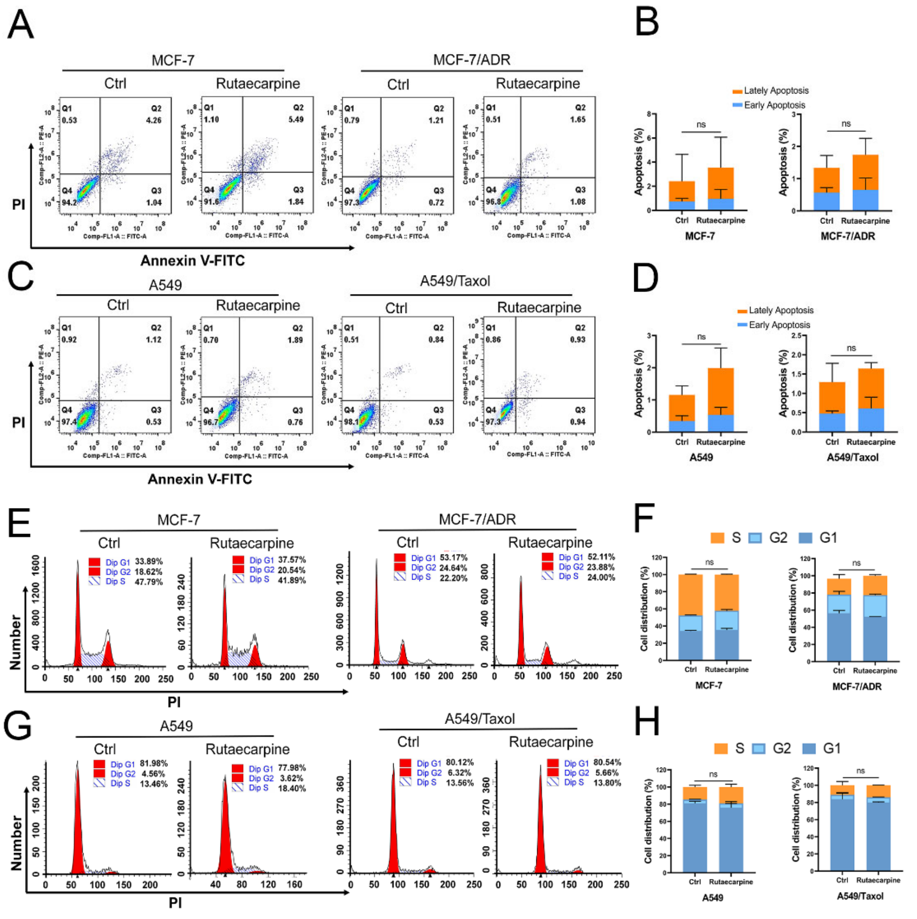

Figure S2. The effect of Rutaecarpine on cells at 20 μM. **(A)** The apoptosis of MCF-7 and MCF-7/ADR cells treated with rutaecarpine (20 μM, 48 h) was determined by Annexin V-FITC/PI double staining assay and **(B)** the percentage of apoptotic cells was analyzed. **(C)** The apoptosis of A549 and A549/Taxol cells treated with rutaecarpine (20 μM, 48 h) was determined by Annexin V-FITC/PI double staining assay and **(D)** the percentage of apoptotic cells was analyzed. **(E)** Cell cycle analysis of MCF-7 and MCF-7/ADR cells treated with rutaecarpine (20 μM, 48 h) was performed by flow cytometry (FCM) and **(F)** the results were statistically analyzed. **(G)** Cell cycle analysis of A549 and A549/Taxol cells treated with rutaecarpine (20 μM, 48 h) was performed by FCM and **(H)** the results were statistically analyzed. All data are expressed as mean ± SD, representative of three independent experiments. ns, not significant.

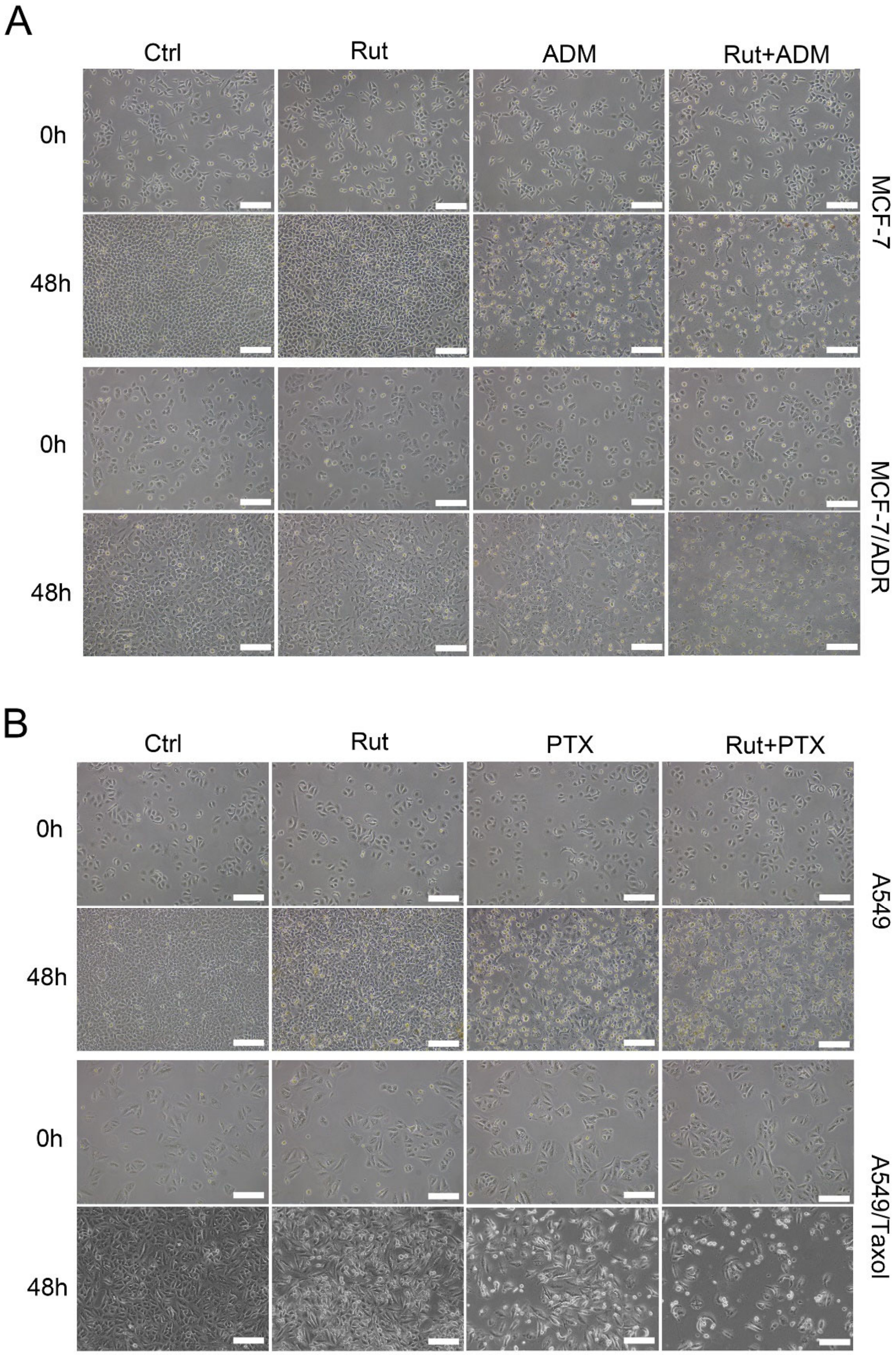

Figure S3. The effects of Rutaecarpine combined with anticancer drugs on the apoptosis of parental and ABCB1-overexpressing cells. **(A-B)** The morphological changes of cells were observed by microscope as shown. Various treatments were given as follows: MCF-7 cells were treated with 20 μM Rutaecarpine and 500 nM adriamycin alone or in combination for 48 h. MCF-7/ADR cells were treated with 20 μM Rutaecarpine and 5 μM adriamycin alone or in combination for 48 h. A549 cells were treated with 20 μM Rutaecarpine and 500 nM paclitaxel alone or in combination for 48 h. A549/Taxol cells were treated with 20 μM Rutaecarpine and 5 μM paclitaxel alone or in combination for 48 h. Scale bar, 200 μm.

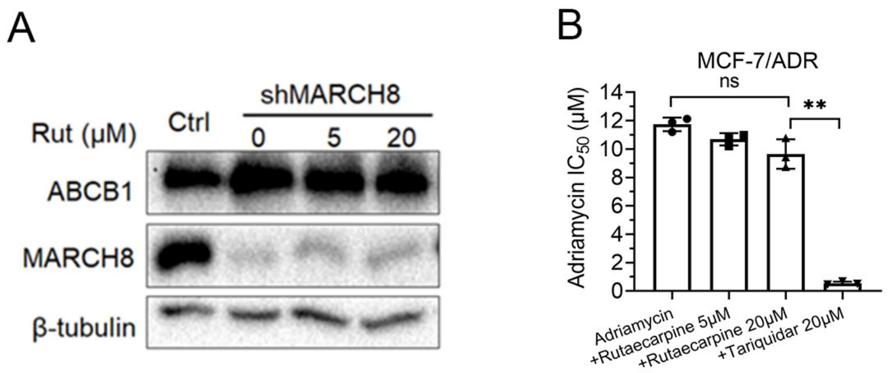

Figure S4. Knockdown of MARCH8 partially prevented the reversal effect of Rutaecarpine on ABCB1. **(A)** ABCB1 expression in MARCH8 knockdown MCF-7/ADR cells incubated with 0, 5, 20 μM of Rutaecarpine for 48 hours was detected by western blotting. **(B)** The effects of Rutaecarpine on the IC_{50} values of adriamycin in MARCH8 knockdown MCF-7/ADR cells. Data are expressed as mean ± SD, representative of three independent experiments. ns, not significant. ** $p < 0.01$.
